# Cryptic Circulation and Co-Infections of Endemic Human Coronaviruses During the First Years of the COVID-19 Pandemic in Brazil

**DOI:** 10.3390/arm93060055

**Published:** 2025-12-05

**Authors:** Ana Karolina Mendes Moreno, Rajiv Gandhi Gopalsamy, Lucas Alves da Mota Santana, Marina dos Santos Barreto, Pedro Henrique Macedo Moura, Deise Maria Rego Rodrigues Silva, Túlio César Rodrigues Leite, Camila de Paula Dias, Breno de Melo Silva, Lysandro Pinto Borges, Ricardo Lemes Gonçalves

**Affiliations:** 1 Institute of Biological Sciences, Federal University of Goiás, Goiânia 74690-900, Goiás, Brazil; 2 Division of Phytochemistry and Drug-Design, Department of Biosciences, Rajagiri College of Social Sciences (Autonomous), Kochi 683104, Kerala, India; 3 Postgraduate Program of Health Sciences (PPGCS), Campus Prof. João Cardoso Nascimento, Federal University of Sergipe, Aracaju 49060-108, Sergipe, Brazil; 4 Department of Dentistry, Federal University of Sergipe, Aracaju 49060-108, Sergipe, Brazil; 5 Postgraduate Program of Pharmaceutical Sciences (PPGCF), Federal University of Sergipe, São Cristóvão 49010-000, Sergipe, Brazil; 6 Laboratory of Biology and Technology of Microorganisms, Department of Biological Sciences, Federal University of Ouro Preto, Ouro Preto 35400-000, Minas Gerais, Brazil; 7 Department of Pharmacy, Federal University of Sergipe, São Cristóvão 49010-000, Sergipe, Brazil

**Keywords:** human coronaviruses (HCoV), co-infection, epidemiology, viral surveillance, SARS-CoV-2

## Abstract

**Highlights:**

**What are the main findings?**

- HCoV-NL63 was the most prevalent species among the HCoVs detected.
- More than 25% of HCoV patients were co-infected with at least one other respiratory pathogen.

**What are the implications of the main findings?**

- HCoVs were circulating during COVID-19 pandemic.
- Coronaviruses can be involved in multi-pathogen infections.

**Abstract:**

During the COVID-19 pandemic, the global focus on SARS-CoV-2 overshadowed the epidemiology of other respiratory pathogens. This study aimed to characterize the circulation of endemic human coronaviruses (HCoVs) in Brazil. We retrospectively analyzed results from 22,472 PCR tests for HCoVs (from 5183 patients) and 601,278 tests for SARS-CoV-2 (from 475,856 patients) between November 2019 and June 2021. HCoVs were detected in 160 patients (3.09%), with HCoV-NL63 as the most frequent species. HCoV circulation was intermittent, with positivity peaks up to 4% but also periods of up to six months with an absence of detections in 2020, contrasting with the sustained high positivity of SARS-CoV-2 (22.37%). Co-infections were frequent: 26.25% of HCoV-positive patients were co-infected with at least one other respiratory pathogen, most commonly Rhinovirus/Enterovirus, and cases involving up to five pathogens were observed, seven patients had co-infections between HCoVs and SARS-CoV-2. These findings reveal the persistent, often cryptic, circulation of HCoVs during the pandemic and highlight their role as key components in complex multi-pathogen infections. This underscores the critical importance of implementing comprehensive molecular diagnostic panels in routine respiratory surveillance to ensure accurate etiology, guide appropriate clinical management, and fully assess the public health burden of non-SARS-CoV-2 coronaviruses.

## 1. Introduction

The coronavirus disease 2019 (COVID-19) epidemic continues to pose a significant global public health challenge due to its emerging variants, high transmissibility, and mortality rates [1,2,3]. In addition to severe acute respiratory syndrome coronavirus 2 (SARS-CoV-2), several other human coronaviruses (HCoVs) have emerged over time. These include SARS-CoV-1, which caused the 2002/2003 outbreak in China, and the MERS-CoV, first documented in 2012 in Saudi Arabia [4,5]. Moreover, four endemic HCoVs with lower pathogenic potential, namely 229E and OC43 discovered in the 1960s, and NL63 and HKU1, identified between 2004 and 2005, are widely distributed globally [6,7,8,9,10,11].

These endemic HCoVs are responsible for nearly one-third of common colds in humans and are frequently associated with mild to moderate respiratory illnesses, though severe cases have been reported in immunocompromised adults and children [12,13]. Importantly, differences in severity and mortality have been observed, with studies reporting higher mortality rates for HCoV-229E compared to HCoV-OC43 [14]. HCoV symptoms often overlap with those of other respiratory pathogens, such as SARS-CoV-2, influenza, adenovirus and rhino/enterovirus, and include cough, nasal discharge, sputum production, difficulty breathing, pneumonia, fever, headaches, and muscle aches [15,16,17,18]. These overlapping clinical manifestations, combined with limited molecular diagnostics, hinder epidemiological surveillance and underscore the need for precise diagnosis to inform appropriate treatment and targeted control strategies, particularly in the post-COVID-19 pandemic era.

HCoVs NL63, OC43, 229E, and HKU1 are globally distributed, although their frequency varies in different regions and times of the year [19]. Studies conducted before the pandemic showed detection rates of endemic HCoVs ranging from 1.2% to 16% in samples, varying between population groups and countries [14,20,21,22,23,24,25,26,27]. A more recent study in a Brazilian cohort of healthcare professionals during the COVID-19 pandemic showed a positivity rate of 5.6% for HCoVs and 33% for SARS-CoV-2 [28]. Other studies have demonstrated the circulation of HCoVs in different populations in the pandemic, including coinfections between HCoVs and SARS-CoV-2 [17,29,30,31,32]. However, data on the prevalence and circulation of common HCoVs during the COVID-19 pandemic have remained limited, as most surveillance efforts were directed toward SARS-CoV-2 [17]. This is even more pronounced in low- and middle-income countries such as Brazil, highlighting the need for continued surveillance in these regions.

Our retrospective study aimed to characterize the circulation dynamics of HCoVs and SARS-CoV-2 infections during the first and second peaks of the pandemic in Brazil. We analyzed a total of 856,762 patient records from November 2019 to June 2021, originating from the COVID-19 Data Sharing/BR database, to describe viral prevalence, demographic factors, and detect co-infections between HCoVs and other pathogens, including SARS-CoV-2. This analysis helps fill knowledge gaps and provide important benchmarks for enhancing public health strategies by elucidating the dynamics of co-circulation and interaction among these viruses.

## 2. Materials and Methods

The data for the study were obtained from the COVID-19 Data Sharing/BR repository (available at https://repositoriodatasharingfapesp.uspdigital.usp.br/ (accessed on 9 September of 2021)). The repository is an initiative of the São Paulo Research Foundation (FAPESP) in collaboration with five hospitals in Brazil: Hospital Israelita Albert Einstein, Instituto Fleury, Hospital Sírio-Libanês, Beneficência Portuguesa de São Paulo and Hospital das Clínicas da Faculdade de Medicina da Universidade de São Paulo. It provides clinical and laboratory test data from 856,762 anonymous patients who underwent COVID-19 tests between November 2019 and June 2021. The repository offers detailed information on patient visits to the hospitals and the tests conducted. In addition to the anonymized patient identifiers, the dataset contains information on the type and date of each test, date, the specific analytes measured, the results, and the corresponding reference values. Because the dataset is provided in Portuguese (the official language of Brazil), all filtering steps used the original Portuguese terms, with English translations added in brackets. The data are available in the repository in .csv format. We performed all screening analyses using R software (version 4.1.1) [33].

To identify the tests performed for HCoV detection, we filtered the data for each hospital, selecting tests targeting the four HCoV (HCoV-NL63, HCoV-HKU1, HCoV-229E, and HCoV-OC43). Specifically, we filtered the rows in the “teste” (test) and “analito” (analyte) columns that contained the following strings: “HKU1”, “OC43”, “NL63” or “229E”. These terms were used to identify and select tests specific to HCoV detection. Then, we checked if all the unique terms in the two columns corresponded to tests for HCoV detection. Only tests utilizing the polymerase chain reaction (PCR) were retained for further analysis.

For the screening of SARS-CoV-2 detection tests, we followed a similar approach. First, we identified unique terms in the “exame” (exam) and “analito” (analyte) columns that could be associated with SARS-CoV-2. Based on these terms, we filtered the rows containing the following strings: “SARS-CoV-2” or “COVID-19” or “ncov” or “novo corona”. These terms were used to identify and select tests specific to SARS-CoV-2 detection. Subsequently, we selected only rows containing the strings “pcr” or “teste molecular” (molecular test) in the “exame” (exam) or “analito” (analyte) columns, ensuring that only PCR-based tests were considered. To ensure an accurate count of all tests performed, we verified that all unique terms identified in the screening represented 100% of the tests for SARS-CoV-2 and HCoV detection. Duplicate records with identical patient ID, attendance ID (when available), test date, test name, analyte, result, and reference fields were removed to retain only unique test entries.

After screening the HCoVs and SARS-CoV-2 detection tests, we selected the tests with positive results. A test was considered positive for HCoVs and SARS-CoV-2 if the terms ‘resultado’ (result) column contained the terms ‘detectado’ (detected) or ‘detectável’ (detectable) or ‘positivo’ (positive). Thus, it was possible to quantify the positive tests for the four HCoVs and SARS-CoV-2, as well as the number of positive patients. We investigated the incidence of viral coinfections in patients with positive tests for at least one of the HCoVs. Using the anonymous patient identifier, we screened the tests performed at the hospitals. We searched only for pathogen detection tests conducted using PCR. Therefore, we selected tests containing the terms ‘pcr’ or ‘teste molecular’ in the “teste” (test) or “analito” (analyte) columns. Subsequently, we selected only tests with positive results in the “resultado” (result) column, using the terms ‘detectado’ or ‘detectável’ or ‘positivo’. We investigated the incidence of viral coinfections in patients with positive tests for at least one of the HCoVs. Finally, we collected demographic data from patients positive for HCoVs and SARS-CoV-2. The demographic data include sex (female or male), year of birth, and state of residence. Based on the patient’s year of birth, we calculated their proximal age. Patients for whom year of birth or residence were suppressed in the repository for anonymization purposes were excluded from the demographic analysis.

Statistical analyses were performed using GraphPad Prism 8.0 (GraphPad Software, San Diego, CA, USA) and R software [33]. Normality of monthly infection positivity rates for endemic HCoVs (OC43, HKU1, 229E, NL63) and SARS-CoV-2 was first assessed via Shapiro–Wilk testing (α = 0.05). Given non-parametric distributions (* *p* * < 0.05 for both cohorts), we applied the Mann–Whitney U tests to compare median positivity rates and distribution disparities between viral groups (February 2020–May 2021), rejecting the null hypothesis of equal medians at * *p* * < 0.05. Spearman rank-order correlation (ρ) to quantify directionality and strength of temporal associations in monthly case trajectories, with |ρ| > 0.5 defined as a strong correlation.

## 3. Results

**Figure 1:**
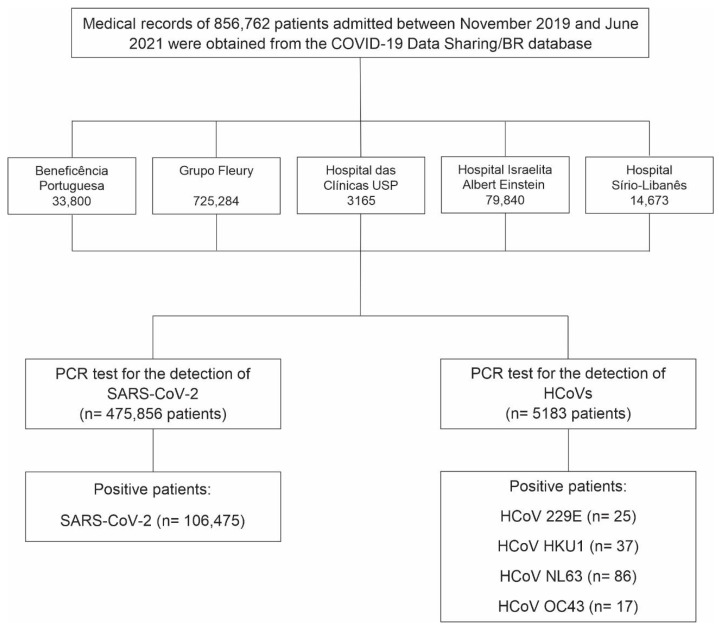
Flowchart showing the number of patients tested and those who tested positive for the four endemic human coronaviruses (HCoVs) and SARS-CoV-2 between November 2019 and June 2021. Data were obtained from PCR screening tests conducted in five Brazilian hospitals and made available by the COVID-19 Data Sharing/BR repository.

**Figure 2:**
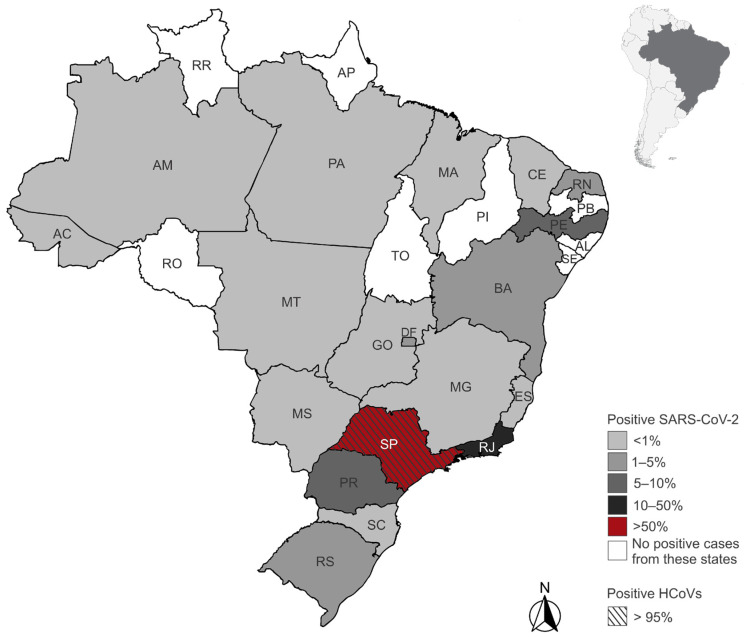
Geographic distribution of endemic human coronaviruses (HCoVs) and SARS-CoV-2 positive patients by state of residence in Brazil. Shades of gray and red represent the proportion of positive cases among all tested individuals in each state. The state of São Paulo is highlighted with diagonal stripes to indicate the presence of positive cases for HCoVs. States in white had no positive SARS-CoV-2 cases reported in the dataset. State acronyms: AC (Acre), AL (Alagoas), AM (Amazonas), AP (Amapá), BA (Bahia), CE (Ceará), DF (Distrito Federal), ES (Espírito Santo), GO (Goiás), MA (Maranhão), MG (Minas Gerais), MS (Mato Grosso do Sul), MT (Mato Grosso), PA (Pará), PB (Paraíba), PE (Pernambuco), PI (Piauí), PR (Paraná), RJ (Rio de Janeiro), RN (Rio Grande do Norte), RO (Rondônia), RR (Roraima), RS (Rio Grande do Sul), SC (Santa Catarina), SE (Sergipe), SP (São Paulo), TO (Tocantins).

We identified 5183 patients who underwent PCR testing for HCoVs between November 2019 and June 2021, of whom 160 (3.09%) tested positive for at least one HCoV (Figure 1). The majority of positive patients were female (*n* = 82), with a mean age of 39.8 years (range: 2 to 89 years), while male patients (*n* = 78) had a mean age of 39 years (range: 3 to 86 years). Among the positive patients, 30 were under 17 years old, with an equal distribution between the sexes. In the 18- to 64-year age group, 110 patients were observed, comprising 56 females and 54 males. In the age group above 65 years, we identified 20 patients, comprising 11 females and 9 males. Detailed demographic characteristics by HCoV type and sex are presented in Table 1. All HCoV-infected patients with available residence data lived in the state of São Paulo (96.9%) (Figure 2). Five patients (3.1%) had their state of residence data anonymized and were excluded from the geographic analysis.

Additionally, we identified 475,856 patients who underwent PCR testing for SARS-CoV-2 between November 2019 and June 2021, of whom 106,475 (22.37%) tested positive (Figure 1-Flowchart). Approximately 51% of the positive patients were female (*n* = 54,681), with a mean age of approximately 42 years for both sexes. The age range was from <1 to 90 years. The majority of cases (84.6%) occurred in individuals aged 18 to 64 years, while 5.5% were under 18 years old and 9.9% were aged 65 years or older. Detailed demographic characteristics by sex are presented in Table 1. We excluded 866 positive patients whose year of birth and/or sex data were anonymized. Most SARS-CoV-2-infected patients resided in São Paulo (59.6%) and Rio de Janeiro (18.2%) states. Patients from 18 other Brazilian states and the Federal District were also identified (Figure 2). Among the positive patients, 1510 (1.4%) had their state of residence data anonymized and were excluded from the geographic analysis.

**Figure 3:**
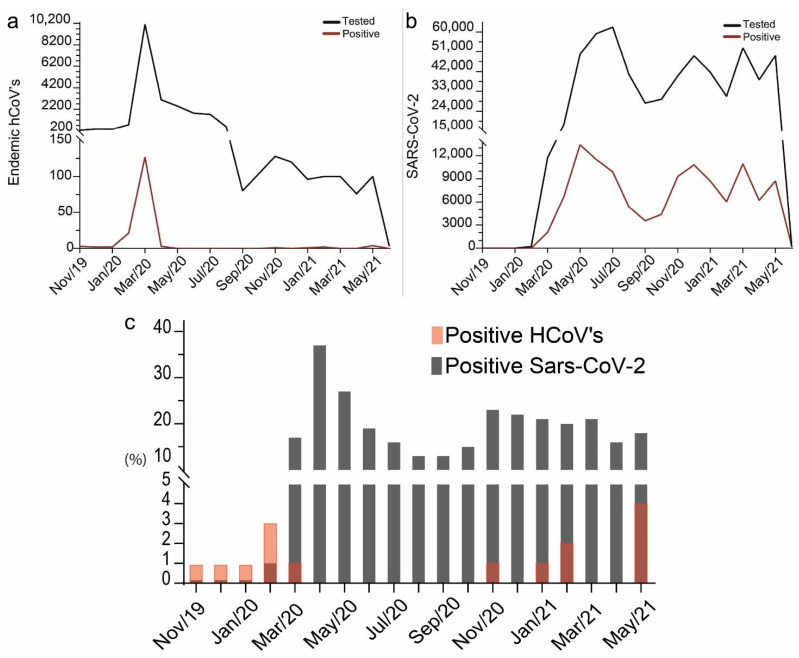
Test rate and positive test rate for (**a**) HCoVs and (**b**) SARS-CoV-2 between November 2019 and June 2021. Black lines represent tests, and red lines represent positive tests. (**c**) Percentage of positive cases relative to tests performed in each month for the four HCoVs and SARS-CoV-2. Gray represents the percentage of positives for HCoVs and transparent red represents the percentage of positives for SARS-CoV-2. A darker red shade results from the red transparency layer overlapping the grey bars.

Between November 2019 and June 2021, 22,472 PCR tests for HCoV detection were conducted. Most originated from hospital units (96.4%), with a smaller proportion from laboratory units (2%) and intensive care units (0.8%). Testing was predominantly concentrated in March 2020 (10,124 tests; 45%), followed by April (3080 tests; 13%) and May (2504 tests; 11%) of 2020 (Figure 3a). The highest number of positive cases for HCoVs was observed in March 2020 (*n* = 127). PCR testing for SARS-CoV-2 began in February 2020, totaling 601,278 tests by June 2021 (Figure 3b). The majority of the test originated from laboratory services (79%), hospital units (17.7%) and emergency department (1.17%), with other origins representing a minor fraction. The months of June and July 2020 recorded the highest testing volumes, with 59,243 (9.8%) and 62,189 (10.3%) tests, respectively (Figure 3b). Testing peaks were also observed in December 2020 (49,125 tests), March (52,782), and May (49,211) of 2021 (Figure 3b). Positivity rates for SARS-CoV-2 reached their highest values in May, June, and December 2020 (13,406, 11,494, and 10,814 positive cases, respectively) and March 2021 (10,938 positive cases) (Figure 3b).

The percentage of positive tests for HCoVs relative to the total tests ranged from 0% to 4% during the analyzed period (Figure 3c). The highest rates were observed in February 2020 (2.7%) and May 2021 (4%). Between May and October 2020, no positive cases of HCoVs were recorded, as well as in December 2020, March, and April 2021. For SARS-CoV-2, the percentage of positive tests ranged from 1% to 37.2%. The lowest rate was observed at the beginning of testing, in February 2020 (1.4%), while the highest percentages occurred in April and May 2020, with 37.2% and 26.9%, respectively. After this period, the positivity rates decreased, but then rose again between November 2020 and March 2021, with values exceeding 19.5%. July 2021 was not included in the analysis, as the available data did not represent the full month.

**Figure 4:**
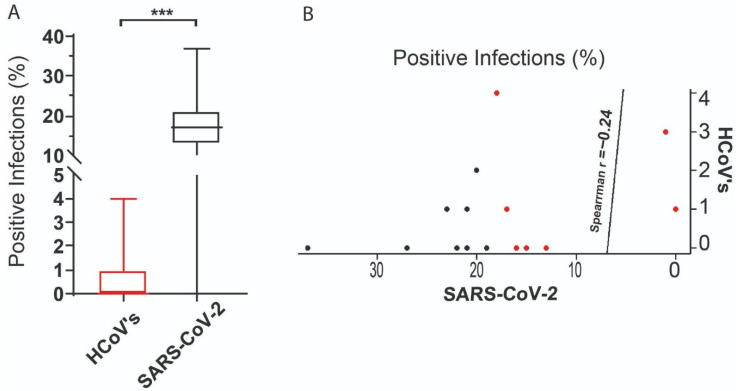
Statistical analysis. (**A**) Comparison and correlation of the distribution of positive HCoV and SARS-CoV-2 tests between February 2020 and May 2021. The boxes represent the interquartile range (IQR) of the percentage of positive infections. The line inside the box refers to the median. The statistical difference observed between the variation in positive cases of endemic HCoV and SARS-CoV-2 is represented by *** = *p* < 0.0001. (**B**) The weak negative correlation between the distribution of positive cases of endemic HCoV and SARS-CoV-2 is represented by the correlation coefficient rho = −0.24. The red dots refer the HCoV’s and the black dots to the SARS-CoV-2 infections.

Statistical analysis of monthly surveillance data (February 2020–May 2021) revealed non-normal distributions for both HCoVs (OC43, HKU1, 229E, NL63) and SARS-CoV-2 cases (Shapiro–Wilk *p* < 0.01). Crucially, Mann–Whitney U testing revealed a divergence in infection trajectories (*** *p* < 0.0001; Figure 4A), with endemic HCoV incidence declining as SARS-CoV-2 established dominance. Temporal mapping identified inverse prevalence patterns: The SARS-CoV-2 surge (e.g., Gamma variant peak in Q1 2021) coincided with HCoV suppression to <25% of pre-pandemic baselines. Quantification via Spearman correlation confirmed a significant but weak negative association (ρ = −0.24; *p* = 0.02; Figure 4B), indicating incomplete niche displacement, which may be a pattern consistent with transient viral interference rather than permanent exclusion.

Among patients positive for HCoVs, 42 (26.25%) had coinfections with eleven pathogens, including SARS-CoV-2 (Table 2). One patient was diagnosed with five simultaneous infections (HCoV-229E, HCoV-OC43, influenza type A, human parainfluenza virus type 3, and Chlamydophila pneumoniae). Six other patients had coinfections with more than two pathogens (Table 2). All multi-pathogen coinfection events occurred in the state of São Paulo.

**Figure 5:**
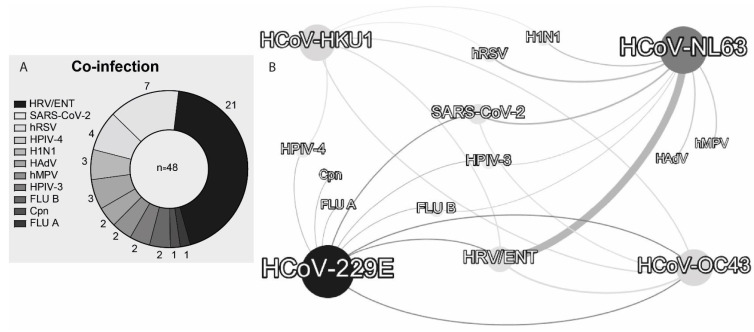
(**A**) Frequency of pathogens identified in association with at least one of the four HCoVs and (**B**) interaction network of HCoVs with other pathogens observed in HCoV-positive patients. The width of the line is proportional to the number of associations found between the pathogens. Abbreviations follow Table 3.

In total, 48 associations between HCoVs and other pathogens were identified (Table 3): human rhinovirus/enterovirus (HRV/ENT) (*n* = 21), SARS-CoV-2 (*n* = 7), human respiratory syncytial virus (HRSV) (*n* = 4), human parainfluenza viruses type 4 (HPIV-4) (*n* = 3), influenza A (FLU-A/H1N1) (*n* = 3), human adenovirus (HAdV) (*n* = 2), human metapneumovirus (HMPV) (*n* = 2), human parainfluenza viruses type 3 (HPIV-3) (*n* = 2), influenza B (FLU-B) (*n* = 2), Chlamydophila pneumoniae (Cpn) (*n* = 1), and influenza a (FLU-A) (*n* = 1) (Figure 5A). HCoV-229E and HCoV-NL63 showed eight associations with other pathogens, while HCoV-HKU1 and HCoV-OC43 showed five and four associations, respectively (Figure 5A). The most frequent association was between HCoV-NL63 and rhinovirus/enterovirus (*n* = 14). The SARS-CoV-2 was associated with three HCoVs, with three associations with HCoV-NL63, two with HCoV-OC43, and two with HCoV-229E. HCoV-OC43 was also associated with two other HCoVs: HCoV-229E and HCoV-HKU1 (Figure 5B).

## 4. Discussion

In this study, we describe the prevalence of infection with four endemic human coronaviruses (HCoV-NL63, HCoV-HKU1, HCoV-229E, and HCoV-OC43) and SARS-CoV-2 in 5183 and 475,856 patients, respectively, who underwent RT-PCR testing in five Brazilian hospitals between 2019 and 2021, pre- and during the COVID-19 pandemic. We observed that HCoV was detected in 3.09% of patients and SARS-CoV-2 was detected in 22.37% of patients. We detected all four HCoV species, with HCoV-NL63 being the most frequent. Moreover, both HCoVs and SARS-CoV-2 were found across all age groups, with no differences by sex, although SARS-CoV-2 was more commonly detected in male patients. We also identified the main patterns of co-infection between HCoVs and other respiratory viruses, which include SARS-CoV-2. Importantly, our study is among the few that have assessed the prevalence of circulating HCoVs in a tropical, developing country during the COVID-19 pandemic using molecular testing. This finding underscores the persistent circulation of HCoVs, even during periods of intense SARS-CoV-2 transmission, and highlights the importance of comprehensive surveillance for respiratory viruses.

We also highlight the distinct and uncorrelated temporal dynamics of HCoV and SARS-CoV-2 during the period. The positivity rate for HCoV tests was low, ranging from >1% to 4%, and included a prolonged period of no detections between May and October 2020. This trend was remarkably independent of that observed in the same period for SARS-CoV-2, which presented a positivity rate ranging from >2% in the first month of the virus’s circulation in the country to a rate of 37% in the third month of circulation, maintaining a rate always ranging from 12% to 26% throughout the analyzed months. This highlights that even during the SARS-CoV-2 pandemic, other coronaviruses also circulated with specific and uncorrelated dynamics. There is no correlation in the distribution of cases between the two infections (Figure 4). The dominance of SARS-CoV-2 is likely explained by its greater transmissibility compared to HCoV [9].

An increase in HCoV testing was observed in February 2020, coinciding with the first confirmed SARS-CoV-2 cases in Brazil, with testing peaking the following month [34]. This high testing may have been a consequence of initial diagnostic uncertainty and the lack of specific molecular tests for the new coronavirus. During this initial phase, PCR tests for other common respiratory pathogens functioned as a diagnostic triage tool [29]. This strategy was supported by early reports suggesting that co-infections with SARS-CoV-2 were rare, leading clinicians to use a positive result for a known virus to aid in the diagnosis and management of patients, though this was later contested [29,35]. In the following months, a series of restrictive measures, in addition to the use of masks, were adopted in Brazil to contain the spread of SARS-CoV-2. Studies indicate that these measures contributed to protection against symptomatic novel coronavirus infection, as well as to decreasing the circulation of other respiratory viruses, including HCoV [15,36,37,38]. In our data, we observed a decrease in the number of patients infected with SARS-CoV-2 and an absence of HCoV detections between May and October 2020. A decrease in the number of monthly tests for HCoV accompanied this reduction. In this context, where respiratory diseases such as HCoV and COVID-19 exhibit similar clinical manifestations, reducing testing to achieve more accurate diagnosis poses a challenge especially in developing countries [17]. Notably, studies reported that among patients who tested negative for SARS-CoV-2 during the pandemic, HCoV was the second most common virus detected [30,39].

Our analysis reveals a significant disequilibrium in the co-circulation patterns between endemic HCoVs and SARS-CoV-2 (*p* < 0.0001), underscoring a profound disruption of the respiratory virus landscape. Despite sharing the same familial origin and inducing similar clinical symptoms, we identified only a weak negative correlation (Spearman’s ρ = −0.24) in the prevalence of these viruses. This finding suggests a complex interplay that deviates from simple competitive exclusion, and the observed disequilibrium may be driven by ecological displacement, where SARS-CoV-2 demonstrates superior fitness traits. This includes a substantially higher binding affinity for the angiotensin-converting enzyme 2 (ACE2) receptor, a critical entry factor it shares with other Betacoronaviruses, such as HCoV-NL63, and enhanced environmental stability in aerosols compared to other seasonal coronaviruses like HCoV-229E and HCoV-OC43 [40,41].

In temperate regions, HCoVs and other respiratory viruses typically have seasonal peaks in winter and reduced circulation in summer [21,42]. However, in tropical and subtropical regions, the few available studies indicate more variable HCoV seasonality patterns [43]. Notably, a long-term study in São Paulo state found that, despite general winter activity, different HCoV species exhibited unique circulation peaks throughout the year [20]. Our findings align with this variability, with HCoV peaks in late summer (February 2020/2021: 2.7 and 2%, respectively) and late autumn (May 2021: 4%), but with no records during winter (June–August 2020). The complete absence of cases during the winter of 2020 coincided with the height of the COVID-19 pandemic, when rigorous public health interventions were implemented, contributing to the decrease in the circulation of other respiratory viruses, as previously mentioned, including HCoV. Understanding this dynamic is important, as research suggests that SARS-CoV-2 will become an endemic coronavirus, similar to HCoV [44]. Therefore, establishing broad long-term surveillance in tropical regions is necessary to clarify the post-pandemic seasonality of HCoVs and may serve as a basis for projecting against the seasonality pattern of SARS-CoV-2 [45].

HCoV-NL63 was the most commonly detected species (*n* = 86), a finding observed in previous studies conducted in tropical and subtropical regions, such as Brazil (São Paulo, Rio de Janeiro, and Teresópolis), China (Zhejiang Province, Hong Kong), and also temperate regions, like Japan (Yamagata Province) [20,32,43,46,47,48]. This contrasts with other studies in temperate regions, where HCoV-OC43 was most commonly detected, especially with peaks of circulation in winter [21,49,50,51]. However, the predominant species may vary according to the geographic region, time of year, and type of population sampled. For example, studies in Brazil have shown this variability: Cabeça et al., 2013 [20] observed a predominance of NL63 and OC43 in São Paulo between 2001 and 2010, but the frequency of these species varied across years and seasons; Góes et al., 2020 [22] observed a predominance of HKU1 in a São Paulo slum between 2005 and 2006; Trombetta et al., 2016 [25] reported OC43/HKU1 as most frequent in southern Brazil between 2012 and 2013. We emphasize that continued surveillance is necessary to better understand the variability in the predominance of each circulating HCoV species over time in different geographic regions in the tropics.

Coronaviruses can infect people of all ages, and in our study, HCoV and SARS-CoV-2 were detected in all age groups, with the highest prevalence in adults (18 to <65 years old). Primary HCoV infections commonly occur in children, but our data confirm that infections in adults are not uncommon, which is consistent with other studies showing frequent reinfections throughout life [21,26]. SARS-CoV-2 also had a lower prevalence in children, consistent with data from a systematic review early in the pandemic, which detected a positivity rate of 1% to 5% in children [52]. Furthermore, SARS-CoV-2 and HCoVs can present as asymptomatic disease in children, leading to lower testing rates among these individuals [16,53]. An additional consideration is the potential role of cross-immunity, especially in children. Although immunity to endemic HCoVs does not appear to be sufficient to prevent SARS-CoV-2 infection, a study suggests that it may reduce symptomatic disease, particularly when prior HCoV exposure occurred within one to two years before SARS-CoV-2 infection, given the relatively short-lived nature of HCoV immunity [54,55]. This study also highlights a direct effect of pre-existing antibodies against HCoVs in preventing symptoms in children who subsequently tested positive for SARS-CoV-2 [56]. The distribution of HCoV infection did not differ between sexes, consistent with previous studies [14,32]. This was also the case with SARS-CoV-2 infection rates; however, a higher prevalence was observed among females in our data, likely reflecting their greater representation in the healthcare system and caregiving roles, which increase exposure risk [56]. In contrast, severe outcomes and mortality were more frequent among men [56,57].

Finally, we observed a high rate of HCoV-infected patients with co-infection with other respiratory pathogens, including cases with two or more HCoVs. This is consistent with studies that document high rates of HCoV co-infection with other respiratory pathogens, including co-infections among all four HCoVs [19,20,32,50,58]. The primary viral partners identified were HRV/ENT, followed by SARS-CoV-2. HRV/ENT are frequently reported as co-pathogens with HCoVs and are a major cause of the common cold, particularly in Latin American countries [20,23,24,27,59]. Furthermore, studies report that HCoVs are among the most common co-infections observed in patients with SARS-CoV-2 [29,30]. An association between HCoV co-infection and increased COVID-19 severity has been reported in hospitalized patients, characterized by longer hospital stays and critically elevated C-reactive protein (CRP) levels compared to patients infected only with SARS-CoV-2 [17]. Among HCoVs, NL63 and OC43 showed the highest frequency in co-infections, consistent with previous studies [17,20]. A review indicated that 71% of NL63 infection cases presented co-infection with other respiratory pathogens [60]. Notably, we also report seven cases of multiple co-infections with three to five respiratory pathogens, consistent with other studies that have documented multiple co-infections involving HCoVs [58,61].

Accurately distinguishing the coronavirus species is vital for proper epidemiological control and adequate monitoring of coinfections [62]. Furthermore, it is also important for establishing prevention programs for vulnerable populations with HCoV positivity reaching 21% in slum communities versus 7.6% in the general population [25,52], especially when diagnosis is based only on clinical presentation due to lack of diagnostic testing [63]. Another important strategic point is the early detection of recombinant variants with zoonotic potential [64]. Prioritizing specific molecular tests, combined with integrated genomic surveillance, is thus essential for agile and accurate epidemiological responses to future epidemics [65].

By analyzing RT-PCR data from a large patient sample in a national repository tested for SARS-CoV-2, this study provides a more precise molecular epidemiology of circulating coronaviruses compared with the serological approaches. The COVID-19 Data Sharing/BR database aggregates records primarily from hospitals located in São Paulo state, and most HCoV-positive results in our dataset originated from a major hospital located in that state. Therefore, the findings may not fully represent the national distribution of respiratory virus infections in Brazil. We highlight a significant difference in the reliability of diagnostic identification between the early and intermediate phases of the pandemic. Cross-reactivity in serological tests, particularly between betacoronaviruses (OC43, HKU1) and SARS-CoV-2, significantly compromised diagnostic specificity, leading to overestimation of positivity rates and distortion of crucial epidemiological indicators [66]. A study that revealed the characteristics of tests approved in the first year of the pandemic confirmed that up to 25% of rapid tests produced false positives due to this cross-reactivity [67]. Thus, our study avoids these confounding factors, offering a more reliable assessment of HCoV and SARS-CoV-2 circulation and co-infection.

In conclusion, our findings reveal persistent circulation of HCoVs during the COVID-19 pandemic. To our knowledge, this is the first Brazilian study to evaluate the circulation of HCoVs during the pandemic, reinforcing data and investigations on co-infections during this period. Thus, this study encourages the implementation of comprehensive molecular diagnostic panels and the guidance of appropriate clinical management for healthcare professionals and the population. In addition, it is necessary to focus on the public health impact of non-SARS-CoV-2 coronaviruses.

## Figures and Tables

**Table 1:** Demographic characteristics of patients who tested positive for endemic human coronaviruses (HCoVs) and SARS-CoV-2, stratified by virus type and sex.

Virus	Sex	Positive Cases *n*/N (%)	<18 Years *n* (%)	18–64 Years *n* (%)	≥65 Years *n* (%)	Min Age	Max Age	Mean Age	SD	SEM
HCoV-NL63	M	41/86 (47.7%)	9 (22%)	27 (65.9%)	5 (12.2%)	3	82	37.15	20.84	3.254
HCoV-NL63	F	45/86 (52.3%)	11 (24.4%)	26 (57.8%)	8 (17.8%)	2	89	38.93	24.12	3.595
HCoV-HKU1	M	19/37 (51.4%)	3 (15.8%)	15 (78.9%)	1 (5.3%)	3	82	39.95	19.11	4.385
HCoV-HKU1	F	18/37 (48.6%)	2 (11.1%)	13 (72.2%)	3 (16.7%)	2	72	44.06	19.99	4.711
HCoV-OC43	M	8/17 (47.1%)	2 (25%)	5 (62.5%)	1 (12.5%)	15	73	41.75	22.03	7.789
HCoV-OC43	F	9/17 (52.9%)	0	8 (88.9%)	1 (11.1%)	29	67	41.89	12.64	4.215
HCoV-229E	M	12/25 (48%)	2 (16.7%)	8 (66.7%)	2 (16.7%)	7	86	37.33	23.46	6.774
HCoV-229E	F	13/25 (52%)	2 (15.4%)	11 (84.6%)	0	7	59	36.54	17.73	4.918
SARS-CoV-2	M	50,928/ 105,609 (48.2%)	2952 (5.8%)	43,054 (84.5%)	4922 (9.7%)	0	90	41.98	16.23	0.072
SARS-CoV-2	F	54,681/ 105,609 (51.8%)	2832 (5.2%)	46,274 (84.6%)	5575 (10.2%)	1	90	41.94	16.36	0.07

Positive Cases *n*/N (%): number of positive cases by sex (*n*), total number of individuals tested for each virus (N), and the corresponding percentage. Age groups show the number and percentage of positive patients in each category (<18 years, 18–64 years, and ≥65 years). Min and Max: minimum and maximum age among positive cases. Mean Age: average age of positive cases. SD: standard deviation. SEM: standard error of the mean.

**Table 2:** Multiple coinfections observed in patients with HCoVs.

Coinfections	N Infections	N Cases	Date
HCoV-229E + HCoV-OC43 + Influenza A virus + Human Parainfluenza virus types III + *C. pneumoniae*	5	1	April 2020
HCoV-NL63 + Rhinovirus|Enterovirus + Human Metapneumovirus + Human Respiratory Syncytial Virus	4	1	March 2020
HCoV-NL63 + Rhinovirus|Enterovirus + Human Respiratory Syncytial Virus	3	1	March 2020
HCoV-NL63 + Rhinovirus|Enterovirus + Adenoviruses	3	2	March 2020
HCoV-HKU1 + Rhinovirus|Enterovirus + Human Parainfluenza virus types IV	3	1	March 2020
HCoV-NL63 + Influenza B virus + SARS-CoV-2	3	1	March 2020

**Table 3:** Co-pathogen detection in patients positive for endemic human coronaviruses (HCoVs).

Co-Pathogen ^a^		HKU1 (*n* = 8)	NL63 (*n* = 28)	OC43 (*n* = 9)	229E (*n* = 11)
HAdV		_	2 (7.2)	_	_
HRV/ENT		2 (25)	14 (50.0)	3 (33.4)	2 (18.2)
HPIV-3		_	1 (3.5)	_	1 (9.1)
HPIV-4		2 (25)	_	_	1 (9.1)
SARS-CoV-2		_	3 (10.7)	2 (22.2)	2 (18.2)
H1N1		1 (12.5)	2 (7.2)	_	_
HMPV		_	2 (7.2)	_	_
HRSV		1 (12.5)	3 (10.7)	_	_
FLU A		_	_	_	1 (9.1)
FLU B		_	1 (3.5)	_	1 (9.1)
Cpn		_	_	_	1 (9.1)
HCoV ^b^	HKU1	_	_	2 (22.2)	_
	NL63	_	_	_	_
	OC43	2 (25)	_	_	2 (18.2)
	229E	_	_	2 (22.2)	_

Data are presented as (%) of each group. ^a^ Virus abbreviations used in this table: HAdV (Human adenovirus), HRV/ENT (Human rhinovirus/enterovirus), HPIV-3 (Human parainfluenza virus type 3), HPIV-4 (Human parainfluenza virus type 4), SARS-CoV-2 (Severe acute respiratory syndrome coronavirus 2), H1N1 (Influenza A H1N1/2009), HMPV (Human metapneumovirus), HRSV (Human respiratory syncytial virus), FLU A (Influenza A), FLU B (Influenza B), Cpn (Chlamydophila pneumoniae), HCoV (Human coronavirus). ^b^ Detection of more than one strain of HCoV.

## Data Availability

The availability of the data is rigorously described in the original repository (https://zenodo.org) complies with the Research Data Alliance guidelines for data sharing (https://doi.org/10.15497/rda00052) as adopted by FAPESP and the collaborating institutions. All data were pseudonymized by the respective institutions prior to inclusion in the database. Because no personal or sensitive information was collected, ethical committee approval was not required for this research.
